# Kinetic assay of starvation sensitivity in yeast autophagy mutants allows for the identification of intermediary phenotypes

**DOI:** 10.1186/s13104-019-4545-0

**Published:** 2019-08-14

**Authors:** Candyce M. Sturgeon, Meaghan R. Robinson, Molly C. Penton, Deanna C. Clemmer, Maria A. Trujillo, Ambar U. Khawaja, Verónica A. Segarra

**Affiliations:** 1 Department of Biology, High Point University, One University Parkway, High Point, NC 27268 USA; 2 Present Address: Department of Biological Sciences, University of North Carolina at Charlotte, Charlotte, NC 28223-0001 USA; 3 Present Address: Cystic Fibrosis Center/Marsico Lung Institute, The University of North Carolina at Chapel Hill, Chapel Hill, NC 27599-7248 USA; 4 Present Address: Department of Human Genetics, Johns Hopkins University, Baltimore, MD 21205 USA; 5 International Baccalaureate Program, High Point Central High School, High Point, NC 27262 USA; 6 Present Address: Campus Y Program (Global Gap Year), University of North Carolina at Chapel Hill, Chapel Hill, NC 27599 USA

**Keywords:** Starvation sensitivity, 96-well plate assay, High-throughput starvation sensitivity assay, Autophagy, Budding yeast, Atg27

## Abstract

**Objective:**

A classical method to quantitatively determine the starvation sensitivity phenotype of autophagy mutant budding yeast strains is to starve them for a period of time and then to assess the proportion of cells that retain the ability to form colonies when the availability of nutrients is restored. The readout of this colony-formation assay is generally evaluated after a fixed period of time following the restoration of nutrients, so that it can be considered an endpoint assay. One drawback we have identified is the inability to characterize subtle intermediary phenotypes that are detectable at the molecular level but fail to reach statistical significance in the colony formation experiment. We set out to determine whether a more dynamic measurement of growth during recovery after starvation would increase the sensitivity with which we are able to detect partial loss-of-function phenotypes.

**Results:**

We describe a 96-well plate-based assay to kinetically assess starvation sensitivity in budding yeast that allows for the quantitative detection of very modest starvation sensitivity phenotypes with statistical significance in autophagy mutant yeast strains lacking the *ATG27* gene.

**Electronic supplementary material:**

The online version of this article (10.1186/s13104-019-4545-0) contains supplementary material, which is available to authorized users.

## Introduction

Starvation sensitivity in yeast is classically scored using an assay that starves cells for a desired period of time for the nutrient of interest (usually carbon or nitrogen source) and then plates these starved cells on nutrient-containing medium to determine how many of them have remained viable and retained the ability to form colonies [1, 2]. This colony formation assay is often used to determine the degree of starvation sensitivity of yeast macroautophagy mutants [1].

Macroautophagy, herein referred to as autophagy, is a catabolic process of cellular self-eating that allows eukaryotic cells to recycle nutrients and sustain essential metabolic processes during periods of stress, such as starvation [3]. Upon induction of autophagy, specialized large double-bilayered autophagic vesicles called autophagosomes non-specifically sequester cytoplasmic materials and fuse with the degradative organelle of the cell. In yeast, autophagosomes deliver the sequestered cytoplasmic components to the lumen of the vacuole for degradation and recycling [3]. Vacuolar membrane proteins then mediate the efflux of the nutrients that are generated through the recycling of these degraded components [3]. This overall cellular process is brought about by a group of proteins known as the Autophagy-related proteins or Atg proteins. Mutations of the *ATG* genes coding for these proteins result in autophagic phenotypes such as starvation-sensitivity. Some of the classical cellular assays to probe yeast autophagy mutants for starvation-sensitivity ultimately assess viability on plates or microscopically using vital dyes [1].

Individual *ATG* gene mutants exhibit characteristic degrees of starvation sensitivity depending on the role played by the encoded Atg protein in the process of autophagy. For example, while deletion of the *ATG1* gene (*atg1Δ*) leads to a complete autophagy phenotype that renders the cell unable to induce autophagy [4, 5], the *atg27Δ* mutation merely results in an autophagic flux delay [6, 7].

**Fig. 1:**
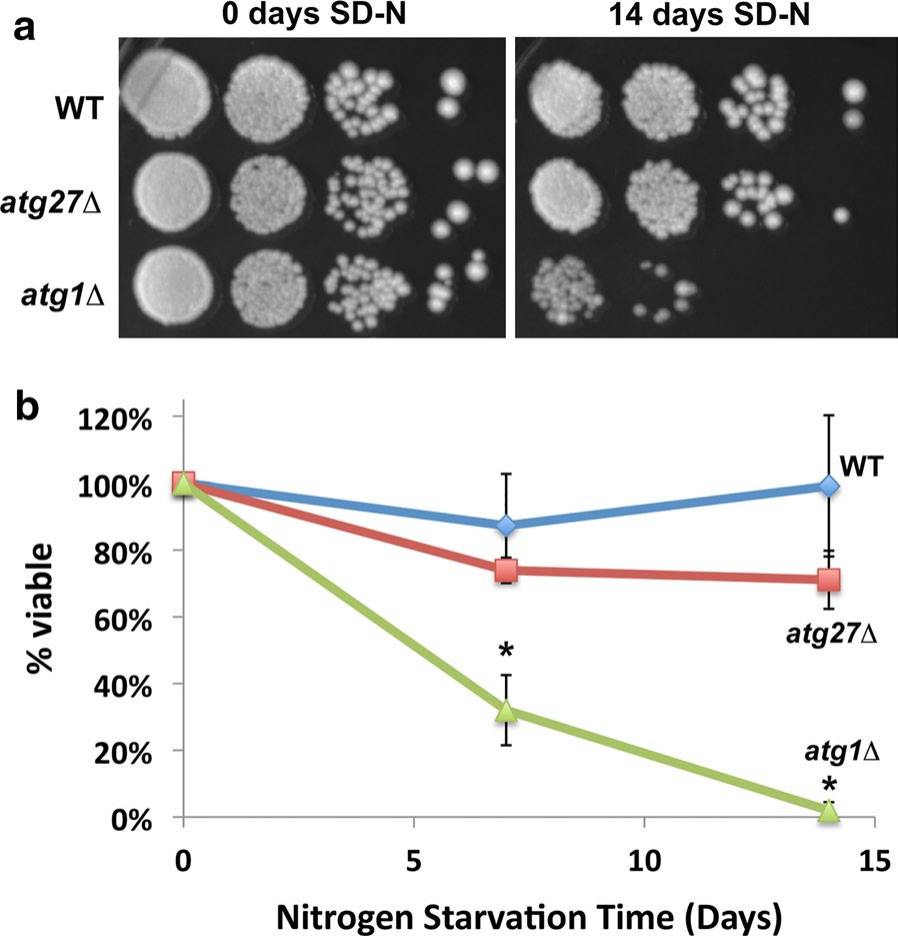
Endpoint methods such as serial dilution and colony formation assays fail to detect a nitrogen-starvation sensitivity phenotype for *atg27Δ* mutants. **a** WT, *atg27Δ,* and *atg1Δ* yeast strains were serially diluted and spotted on YEPD medium after 0 or 14 days of SD-N treatment (nitrogen starvation). The WT and *atg27Δ* strains were indistinguishable from one another despite published biochemical evidence that *atg27Δ* strains have autophagic marker processing and autophagosome formation defects. As expected, *atg1Δ* displayed a strong starvation sensitive phenotype that was easily noticeable when compared to WT. **b** Colony formation units were quantified after SD-N treatment of WT, *atg27Δ,* and *atg1Δ* yeast strains for the indicated period of time. The percentage of colonies produced by the same OD_600_ and volume of culture at day 0, 7, or 14 post-starvation (% viability) was calculated and plotted as a function of nitrogen starvation time (days). No statistically significant difference was observed between the starvation sensitivity of WT and *atg27Δ.* All experiments were carried out at least three times, with at least three technical replicates for each experiment. While this plot was generated using a set of three technical replicates, all experiments displayed the same trends. Error bars indicate standard deviations, and *p*-values less than 0.05 are flagged with one asterisk (*)

These phenotypic characteristics reflect the functions these players have in autophagy: while Atg1 is a protein kinase required for the induction of autophagy, Atg27 is a membrane protein that facilitates (but is not essential for) autophagosome formation [6, 7]. For mutants like *atg1Δ,* starvation sensitivity can be robustly detected even using agar plating methods such as patching or serial dilution assays that have no immediate quantitative readout (4, Fig. 1a). These methods nicely complement biochemical assays that detect events at the molecular level such as defective processing of autophagic markers [6–8]. For mutants with subtle autophagy phenotypes such as *atg27Δ*, agar plating methods do not provide the dynamic range required to accurately measure a starvation sensitivity phenotype. While the aforementioned biochemical methods detect the intermediary autophagy phenotypes of mutants like *atg27Δ* more accurately, there has remained a longstanding need for complementary cellular assays to assess defects in these mutants.

To fill this gap, we set out to design a 96-well plate-based assay to kinetically assess starvation sensitivity in budding yeast that quantitatively detects very modest starvation sensitivity phenotypes with statistical significance in autophagy mutant yeast strains lacking the *ATG27* gene. Similar strategies have been reported by others to increase quantitative detection of drug sensitivity in yeast [9].

## Main text

### Methods

#### Yeast strains

The strains used in this study were obtained from Professor S. Lemmon (Department of Molecular and Cellular Pharmacology, University of Miami Miller School of Medicine, USA) and their provenance and construction was previously described in detail by Segarra et al. and others [7, 10]. The genotype of these strains is as follows: wild type or WT (*MATα leu2 ura3*-*52 trp1 his3*-*Δ200*), *atg27Δ* (*MATα leu2 ura3*-*52 trp1 his3*-*Δ200 atg27Δ::HISMX6*), and *atg1Δ* (*MATα leu2 ura3*-*52 trp1 his3*-*Δ200 atg1Δ::NATMX6*).

#### Yeast media and growth

Standard yeast media and growth conditions were used in all experiments [11]. The medium used to starve cells for nitrogen and induce autophagy was SD-N, consisting of 0.17% yeast nitrogen base without ammonium sulfate and amino acids, and 2% glucose [1].

#### Serial dilution assays

Serial dilution assays were set up using a slightly modified version of a previously described protocol [12]. Briefly, liquid cultures of the yeast of interest were first inoculated and grown to saturation in rich medium (YEPD). Cells were then diluted to an OD_600_ of 0.5, and serial dilutions of 1:10 were spotted on the appropriate solid agar plates before and after nitrogen starvation.

#### Colony formation assays

Colony formation assays were performed as described previously [1, 2].

#### Kinetic or growth curve assays

Growth curve assays to assess starvation sensitivity were performed using a modified version of a previously described protocol [9]. Liquid cultures of the yeast strains of interest were inoculated and grown to saturation in rich medium (YEPD). Cultures were then diluted to an OD_600_ of 0.5 before washing twice and transferring to SD-N starvation medium. At the desired starvation time points (including as a reference or control time point 0 for no starvation), aliquots of the culture of interest were taken and diluted serially (1:10) in rich or complete medium. This provided back nutrients at the indicated time points on a 96-well plate, accommodating 4 technical replicates of each of the three strains tested per dilution per experiment. An absorbance microplate reader (BioTek) was used to track yeast growth at 30 °C in each well of the 96-well plate by measuring OD_600_ every 30 min over a 40-h time period.

### Results

Serial dilution and colony formation assays were initially used to measure starvation sensitivity (Fig. 1). The WT and *atg27Δ* strains were indistinguishable from one another despite published biochemical evidence that *atg27Δ* strains exhibit autophagic marker processing and autophagosome formation defects at the molecular level [6, 7]. As expected, the serial dilution and colony formation assays successfully detected a starvation-sensitivity phenotype for the *atg1Δ* control (Fig. 1).

**Fig. 2:**
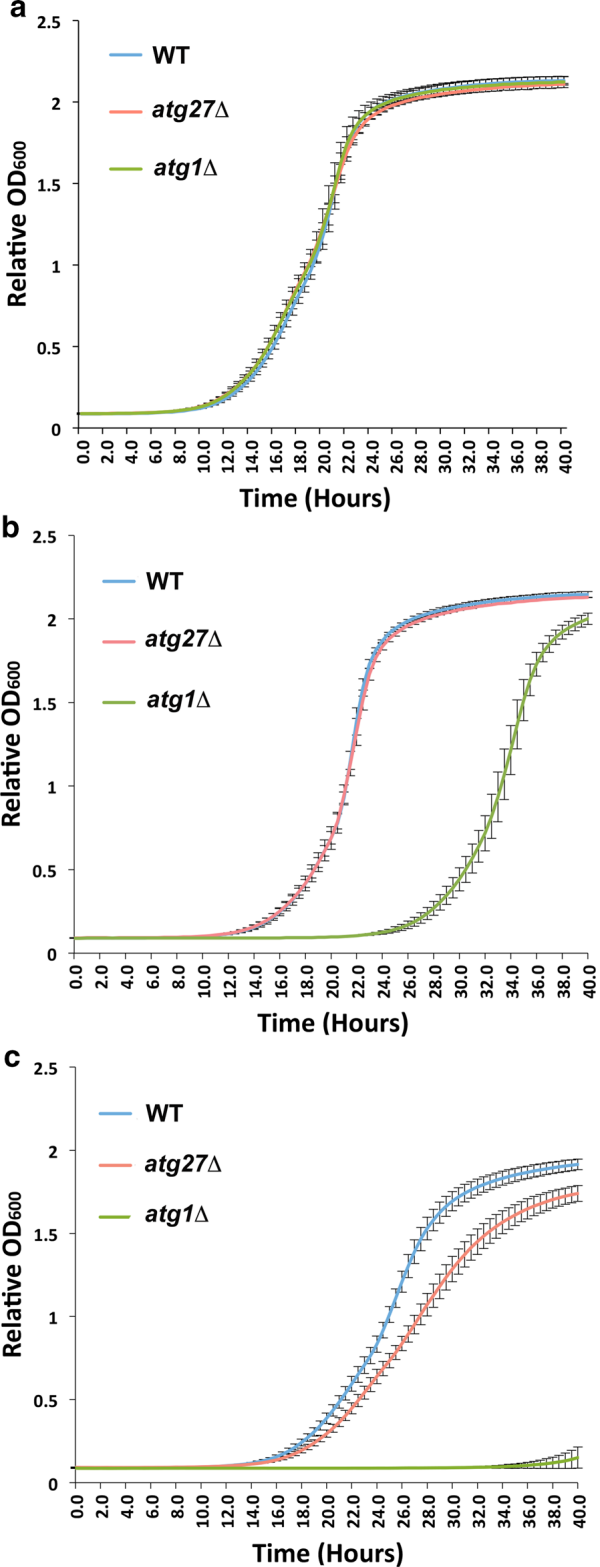
A kinetic method to assess growth after starvation detects a subtle but statistically significant nitrogen-starvation sensitivity phenotype of *atg27Δ* mutants. WT, *atg27Δ*, and *atg1Δ* yeast strains were grown in the presence of nutrients either before nitrogen starvation (**a**), after 7 days of nitrogen starvation (**b**), or after 14 days of nitrogen starvation (**c**). All experiments were carried out at least three times, with at least four technical replicates for each experiment. While plots (growth curves) were generated using a set of four technical replicates, all experiments displayed the same trends. Error bars indicate standard deviations. Corresponding *p*-values are listed in Additional file 1: Table S1 and are highlighted if less than 0.05

A kinetic starvation-sensitivity assay was designed to investigate the possibility of a subtle nitrogen-starvation sensitivity phenotype of the atg27Δ mutant by measuring its OD_600_ in rich liquid medium at a greater number of time points following a period of starvation (Fig. 2). A subtle but statistically significant nitrogen-starvation sensitivity phenotype was detected for the *atg27Δ* mutant following both 7-day and 14-day periods of starvation. Comparison of *p*-values across 80 different time points collected over a 40-h period of recovery after starvation indicated that this intermediary phenotype was detected with greatest significance over the time points coinciding with logarithmic growth (Additional file 1: Table S1). It was not necessarily surprising that subtle phenotypic differences were most detectable during log phase. In addition, this indicated a potential explanation for the failure of previous prepared media plate-based assays to detect the phenotype, in that these longer, endpoint assays provided sufficient time for the *atg27Δ* mutant and its WT counterparts to complete log phase growth and become phenotypically indistinguishable by the time the measurement was made. As expected and as previously described [4], *atg1Δ* displayed a strong starvation sensitive phenotype that was easily noticeable and statistically significant when compared to WT (Fig. 2 and Additional file 1: Table S1). Similar results were obtained for all strains when using rich or complete medium to restore nutrients.

### Discussion

In this report, we describe a 96-well plate-based assay to kinetically assess starvation-sensitivity in budding yeast, allowing for the quantitative detection of very modest phenotypes with statistical significance. We tested this method using strains lacking the *ATG27* gene and exhibiting a modest defect in autophagy. While *atg27Δ* cells following nitrogen starvation appeared equivalent to their wild-type counterparts in traditional colony formation and serial dilution assays (Fig. 1), the kinetic assay detected a subtle but statistically significant defect. This phenotype was consistent with published biochemical evidence that *atg27Δ* strains exhibit diminished autophagic flux [6, 7]. A specific advantage of the kinetic approach was that differences between the WT and mutant strains were most apparent and statistically significant within a narrow range of time points along the growth curve, yet indistinguishable at later time points when control and experimental strains began to reach saturation and slow down (Additional file 1: Table S1). Subtle phenotypes such as this one might be ideally detected in a dynamic assay rather than in an endpoint assay. We note the increasingly widespread use of technologies that support and monitor cellular growth over time in a dynamic way, including in yeast applications such as drug sensitivity screening [9]. We propose that this trend will benefit both low and high throughput studies by detecting a broader spectrum of phenotypes ranging from strong to intermediary.

## Limitations

The experiments described in this manuscript assay cells for their ability to proliferate and grow to contribute to colony formation or an increase in OD_600_ as a function of time, not for whether cells are alive or dead. It is well known that upon starvation for nutrients like nitrogen, cells arrest in a G_1_/G_0_ quiescent state [13]. It is possible that specific mutants could fail to exit this G_1_/G_0_ state and therefore be rendered unable to resume proliferation when nutrients are replenished. If this is the case with a particular mutant of interest, the assays described here would fail to assess accurately the viability of cells after starvation.

## Additional file

**Additional file 1: Table S1.** *p*-values associated with the kinetic starvation sensitivity assays presented on Fig. 2a, b.

## Data Availability

The datasets generated and/or analyzed during this study are available from the corresponding authors on reasonable request.

## Abbreviations

*ATG* genes: autophagy-related genes
Atg proteins: autophagy-related proteins
WT: wild type
SD-N: synthetic dextrose medium without ammonium sulfate or nitrogen source
YEPD: yeast rich medium: yeast extract, peptone, and dextrose
