# Supplementary material for: Kinetic assay of starvation sensitivity in yeast autophagy mutants allows for the identification of intermediary phenotypes
Source: BMC Res Notes. 2019 Aug 14;12:505. doi: 10.1186/s13104-019-4545-0 (PMC6694668; doi:10.1186/s13104-019-4545-0)
Supplement: Supplementary file 1 — Additional file 1: Table S1. p-values associated with the kinetic starvation sensitivity assays presented on Fig. 2a, b. [file 13104_2019_4545_MOESM1_ESM.docx]

**Additional file 1: Table S1.** *p-values associated with the kinetic starvation sensitivity assays presented on Figure 2A and 2B.* *p*-values listed below are highlighted if less than 0.05 or significant. 7d and 14d refer to the starvation periods in days. The times in hours (1^st^ column) correspond to the subsequent growth/recovery time points at which measurements were made.

| **Time** | ***p*-values** | | | |
| --- | --- | --- | --- | --- |
| **Hour** | ***atg27Δ* 7d** | ***atg1Δ* 7d** | ***atg27Δ* 14d** | ***atg1Δ* 14d** |
| 12 | 0.495025346 | 0.001608867 | 0.181690114 | 0.001308304 |
| 12.5 | 0.69188401 | 0.001769836 | 0.035352847 | 0.001241545 |
| 13 | 0.579715109 | 0.00151978 | 0.035081515 | 0.001109953 |
| 13.5 | 0.66965284 | 0.00188741 | 0.015587358 | 0.001025004 |
| 14 | 0.655494235 | 0.001689787 | 0.031776981 | 0.001220598 |
| 14.5 | 0.581785481 | 0.001275677 | 0.019329285 | 0.001374494 |
| 15 | 0.553056295 | 0.00118905 | 0.012008009 | 0.001563178 |
| 15.5 | 0.575049725 | 0.00093509 | 0.011749751 | 0.001422721 |
| 16 | 0.656303412 | 0.000840693 | 0.014300459 | 0.001688828 |
| 16.5 | 0.726300648 | 0.000772135 | 0.009286789 | 0.001377914 |
| 17 | 0.795003396 | 0.000727242 | 0.007716458 | 0.001339783 |
| 17.5 | 0.864504353 | 0.000665434 | 0.005291461 | 0.000993922 |
| 18 | 0.956142969 | 0.00065058 | 0.005032512 | 0.000852615 |
| 18.5 | 0.941756626 | 0.000590157 | 0.005946755 | 0.00068574 |
| 19 | 0.903251512 | 0.000529271 | 0.004737286 | 0.000588049 |
| 19.5 | 0.913334534 | 0.000455502 | 0.004248916 | 0.000478556 |
| 20 | 0.978541923 | 0.000401138 | 0.003947434 | 0.000437214 |
| 20.5 | 0.795587955 | 0.000198699 | 0.004068493 | 0.000386545 |
| 21 | 0.69604465 | 1.99651E-05 | 0.003847917 | 0.000320552 |
| 21.5 | 0.792222111 | 3.89099E-05 | 0.003988348 | 0.000291554 |
| 22 | 0.467581576 | 6.72075E-05 | 0.004523051 | 0.000260753 |
| 22.5 | 0.224223622 | 1.13628E-05 | 0.005286845 | 0.000236294 |
| 23 | 0.106470421 | 1.00959E-06 | 0.006149214 | 0.000199221 |
| 23.5 | 0.071837179 | 7.15917E-08 | 0.005427916 | 0.000150183 |
| 24 | 0.076049703 | 1.33231E-08 | 0.005131478 | 0.000111129 |
| 24.5 | 0.044469856 | 1.87048E-07 | 0.005281191 | 6.78741E-05 |
| 25 | 0.045506388 | 3.78925E-07 | 0.005743778 | 5.5433E-05 |
| 25.5 | 0.040519326 | 6.8996E-07 | 0.006037251 | 6.33323E-05 |
| 26 | 0.047470664 | 1.1541E-06 | 0.005896407 | 7.38366E-05 |
| 26.5 | 0.054513822 | 2.04242E-06 | 0.006307861 | 7.85707E-05 |
| 27 | 0.051037167 | 2.99075E-06 | 0.006142076 | 6.83434E-05 |
| 27.5 | 0.065762707 | 4.70215E-06 | 0.005767319 | 5.50361E-05 |
| 28 | 0.076892769 | 6.85166E-06 | 0.005647799 | 4.58195E-05 |
| 28.5 | 0.070217107 | 9.77671E-06 | 0.005005125 | 3.61919E-05 |
| 29 | 0.101201892 | 1.51797E-05 | 0.005103966 | 3.14391E-05 |
| 29.5 | 0.109336488 | 2.27706E-05 | 0.004726902 | 2.4654E-05 |
| 30 | 0.103385198 | 3.28927E-05 | 0.004625783 | 2.09318E-05 |
| 30.5 | 0.102038118 | 5.02691E-05 | 0.004534675 | 1.75826E-05 |
| 31 | 0.106758903 | 7.54288E-05 | 0.004696637 | 1.53297E-05 |
| 31.5 | 0.13592464 | 0.000109355 | 0.005088874 | 1.31823E-05 |
| 32 | 0.10286424 | 0.000173155 | 0.00539931 | 1.25686E-05 |
| 32.5 | 0.118198655 | 0.000377953 | 0.005377459 | 1.09476E-05 |
| 33 | 0.153772039 | 0.000659397 | 0.005755661 | 1.02571E-05 |
| 33.5 | 0.104101079 | 0.000943124 | 0.005625551 | 9.64859E-06 |
| 34 | 0.089582772 | 0.001220309 | 0.005908921 | 9.45234E-06 |
| 34.5 | 0.105008713 | 0.001961816 | 0.005299407 | 9.21626E-06 |
| 35 | 0.13088079 | 0.002156886 | 0.005646052 | 9.70306E-06 |
| 35.5 | 0.125437114 | 0.001965716 | 0.005980822 | 9.71784E-06 |
| 36 | 0.103843517 | 0.001683387 | 0.006474597 | 1.06762E-05 |
| 36.5 | 0.151671542 | 0.00163309 | 0.006652371 | 1.15589E-05 |
| 37 | 0.118509961 | 0.002092273 | 0.006164726 | 1.40706E-05 |
| 37.5 | 0.150005701 | 0.002331751 | 0.005990181 | 1.56037E-05 |
| 38 | 0.130860587 | 0.003101165 | 0.006251122 | 1.87714E-05 |
| 38.5 | 0.17803142 | 0.003917336 | 0.005999915 | 2.28393E-05 |
| 39 | 0.135215282 | 0.00463634 | 0.005757762 | 2.98581E-05 |
| 39.5 | 0.143299618 | 0.005707004 | 0.006282744 | 3.96217E-05 |
| 40 | 0.1491324 | 0.007178994 | 0.006005968 | 5.36097E-05 |
